# A Treatise on Human Physiology

**Published:** 1859-07

**Authors:** 


### NORTH AMERICAN

### MEDICO-CHIRURGICAL REVIEW.

JULY, 1859.

### Analytical and Critical Reviews.

ART. I.—*A Treatise on Human Physiology; designed for the use of Students and Practitioners of Medicine.* By JOHN C. DALTON, JR., M.D., Professor of Physiology and Microscopic Anatomy in the College of Physicians and Surgeons New York, etc. 8vo., pp. 608. With 254 illustrations. Blanchard & Lea: Philadelphia, 1859.

*Outlines of Physiology.* By JOHN HUGHES BENNETT, M.D., F.R.S.E., Professor of the Institutes of Medicine, and Senior Professor of Clinical Medicine in the University of Edinburgh, etc. 12mo., pp. 247. A. & C. Black: Edinburgh, 1858.

*Cours de Physiologie Comparée. De l'Ontologie ou Étude des Êtres. Leçons professées au Muséum d'Histoire Naturelle.* Par M. FLOURENS, Recueillies et rédigées par CHAS. ROUX. 8vo., pp. 184. J. B. Baillière: Paris, 1856.

*Leçons sur la Physiologie et l'Anatomie Comparée de l'Homme et des Animaux faites a la Faculté des Sciences de Paris.* Par H. MILNE EDWARDS. Tomes 1, 2, 3 et 1re partie de tome 4. Victor Masson: Paris, 1857.

*Cours de Médecine du Collége de France. Leçons sur la Physiologie et la Pathologie du Système Nerveux.* Par M. CLAUDE BERNARD. Tomes 1, 2. J. B. Baillière: Paris, 1858.

THE study of life and its phenomena presupposes the study of living beings—the receptacles or media of life—the instruments or mechanism through which the vital principle is manifested. Reasoning from the known to the unknown, we may, with philosophical propriety, attempt to abstract or isolate this principle, as one factor of a complex problem, and

By J. L. Ap so study it in its physical, chemical, and vital aspects. Practically, however, we must study life in its anatomical relations; in other words, the structure of living beings demands our attention as the inseparable companion of life. The study of function, to be profoundly successful, must always be associated with the examination of structure. The life of an animal or vegetable is manifested by certain active phenomena, which differ as they are exhibited in different parts or organs of that animal or vegetable. These phenomena are in truth the functions of the organs in which they appear; and these organs are peculiar collocations of organic matter through which, under the influence of the vital principle, these functions or life-phenomena are exhibited, and without which the latter could find no expression.

Now anatomy may be studied alone. Its records are written upon the dead body. With biology or physiology it is different. It must be studied in conjunction with anatomy, since through the functions, which are the actions of the organs, our principal knowledge of biology is obtained. The study of anatomy is a preliminary step, though by no means the key to physiology. "In my opinion," very justly says Milne Edwards, in the introduction to his great work now before us, "anatomy and physiology are inseparable parts of one and the same science. Not only are they mutually and necessarily supporting, but they have one object in common, and they should always be associated, therefore, in the mind of every one who, following the example of Aristotle, seeks to understand the *nature of animals.*"

For every living being there is not only a characteristic topographical or regional anatomy, peculiar to the variety of the species to which the creature belongs, but also certain anatomical relations, both specific and generic, by which it is bound to every other being on the surface of the globe, and in virtue of which it is seen to be part of an infinitely diversified idea or plan. So, also, for everything living there is a special or individual physiology—a physiology of the variety of a species, and a comparative or relational physiology, which concerns the species and the genus only. The life-actions of the fish are not identical with those of the reptile, neither is the physiology of these two groups of animals precisely like that of birds or mammals. The herbivora absorb less oxygen, in proportion to the carbonic acid exhaled, than the carnivora. In the ox the process of digestion is more complicated than in the horse.

The law of differentiation, or, in other words, the law of progression, from the heterogeneous or general to the homogeneous or special, is necessarily as applicable to the actions of an organism, whether vegetable or animal, as to its structure. No organism in the whole series of animated nature is isolated; neither are the actions of that organism. Both are interconnected respectively with the forms and actions of all the higher and all the lower individuals of the great organic series.

Among the ancients, the term physiology was synonymous with natural philosophy. With them, a treatise upon the formation of clouds, the precipitation of rain, the absorption of luminous solar rays by the atmosphere, etc. was as much a part of physiology as an essay upon the movements of the heart in animals, or the circulation of the sap in plants. At the present time, however, the term is used in a much more restricted sense. Instead of embracing all nature in its scope, it is now applied, with obvious etymological impropriety, to one part or division only of the great science of nature. Starting systematically, then, we may say that two branches of inquiry claim the attention of the student of nature—First. Physics, (φυσις, *natura,*) or physiology, (φυσις, λογος,) as it ought to be called, the science of nature as a whole. Second. Biology, (βιος, *vita,*) the science of life, or of the so-called animate world. Treviranus* and Foderé were among the first to substitute this latter term for that of physiology. They used it to signify the properties which differentiate organic and inorganic matter. According to Bostock, however, the word "may be considered as applicable rather to the general principles which constitute the theory of the science, than to the descriptive part from which these principles are deduced."† Ducrotay de Blainville employed the words *zoobiology* and *zoobie* indifferently, to express the internal phenomena of the organism in their relation to external conditions.‡ Long before him, however, Darwin applied the term zoonomia to the laws of organic life. Dr. Bennett, in his philosophical *Outlines*, employs the term physiology in the enlarged sense of comprehending the doctrine of life, whether in health or disease, which, he says, would perhaps more properly be denominated biology. Flourens divides physiology into biology, or the study of life proper, and ontology, or the study of living beings. Ontology he divides into neontology and palæontology. Substituting the word biology for physiology, we may designate the physiology of any particular organized body by the term Idiobiology, (ιδιος, βιος,) and the physiology of the species or genus to which that body belongs by the term Biontology, (βιος, οντος.) The standard works on human physiology are so many treatises on Idiobiology. Human Biontology, or the comparative physiology of the races of man, has not yet found an exposition. The nearest approach to it, perhaps, is Boudin's recent valuable and elaborate *Traité de Géographie et de Statistique Médicales, etc.*

* Biologie, oder Philosophie der lebenden Natur für Naturforscher und Aertze.

† Elementary System of Physiology; London, 1836.

‡ Cours de Physiologie Générale et Comparée; Paris, 1833, tome i p. 3.

The nature of the vital fluid; its circulation throughout the body; the manner in which it is aerated or vivified, so to speak, and enabled to run its fertilizing course; the process by which it receives constant and necessary supplies; the manner in which it gives, on the one hand, pabulum to the tissues, and, on the other, certain recremento-excrementitial matters to the various secerning organs; the mysterious process by which the nutritive material is assimilated; the manner in which animal heat is maintained, —such are some of the subjects which it is the province of Idiobiology to investigate.

Biontology, embracing, as it does, the transcendental or strictly philosophical in physiology, occupies wider, and, if possible, higher ground. It treats of the ovum; its origin and developmental career from the general to the special, or, to use the language of Von Baer, from the homogeneous to the heterogeneous; the laws of formation and deformation; the doctrine of monstrosities, of arrests of development, etc. It investigates the creation and extinction of species and genera, the order of their succession in time, and their distribution in space; their fixity or mutability; the amount of life upon the globe, and the laws governing that amount; the specific and generic differences and relations of existing animals and plants. It goes still further, and inquires into the functional history of the ancient Flora and Fauna, whose fossil remains are the only records of that history. Lastly, it concerns itself with the races of men, and attempts to solve the obscure and perplexing problems of sociology and ethnology—discussing questions of social ethics, of moral and educational interests, and essaying to determine by its own investigations, whether man be one or many, whether the human family be monogenous or polygenous in origin. From this summary, it will be seen that Biontology subdivides itself into the sciences of embryology, organic morphology, teratology, neontology, palæontology and ethnology. To these, psychology or the science of mind has also been added. And here it is that physiology touches upon the domain of the metaphysician and the theologian.

Thus it appears that biology is at once a general and a special science; for the functions, which are simply the actions or phenomena springing from the union of the life-principle and organic structure, separate naturally into three great classes: the nutritive, vegetative, or assimilative, those concerning the individual, and whose great object is to preserve the structural integrity of the organism in a healthy condition; the reproductive, which provides for the preservation of the race, species, or genus, by generating new organisms to take the place of the old, the former exhibiting all the specific peculiarities of the latter; and, finally, superadded to these in the animal, the functions of relation or of animal life, by which the animal is brought into relation with surrounding objects, and by means of which it is protected to a certain extent from destructive influences without.

Such is the classification very correctly adopted by Professor Dalton in his *Treatise on Human Physiology.* This work he divides into three sections. The first is devoted to nutrition, under which head he discusses, successively, the proximate principles, food, digestion, absorption, the bile, formation of sugar in the liver, the spleen, the blood, respiration, animal heat, the circulation, secretion and excretion. All this is embraced in 275 pages. In the second section, which occupies 120 pages, is considered the physiology of the nervous system. This section comprises six chapters, which treat respectively of the general character and functions of the nervous system; of nervous irritability, and its mode of action; of the spinal cord, the brain, the cranial nerves, and the great sympathetic system. Reproduction falls into section third, and takes up 164 pages. This section is divided into eighteen chapters, which are severally devoted to the consideration of the following subjects: The nature of reproduction, and the origin of plants and animals; sexual generation, and the mode of its accomplishment; the egg, and the female organs of generation; the spermatic fluid, and the male organs of generation; periodical ovulation, and the function of menstruation; the corpus luteum of menstruation and pregnancy; the development of the impregnated egg; the umbilical vesicle; the amnion and allantois, and the development of the chick; the development of the egg in the human species, and the formation of the chorion; the development of the uterine mucous membrane, and the formation of the decidua; the placenta; the discharge of the ovum, and involution of the uterus; the development of the nervous system, organs of sense, skeleton, and limbs; development of the alimentary canal and its appendages; development of the kidneys, wolffian bodies, and internal organs of generation; development of the circulatory apparatus; development of the body after birth. From this recapitulation of its contents, and the space devoted to each section, it will be seen that this work is in reality a condensed treatise or essay upon reproduction, accompanied with a very general survey of the physiology of organic and animal life; a survey, it must be said, altogether too purely physiological to meet the daily wants of the practitioner of medicine; though very well adapted, for several reasons presently to be stated, as a primary work, to be placed in the hands of the student who is just commencing his physiological studies.

Professor Bennett's *Outline of Physiological Science* is also divided into three parts. The first treats of histological physiology; the second of normal or healthy physiology, and the third of pathological physiology.

Though much smaller, and still more deficient in details than the work of Professor Dalton, it is certainly more practical in its conception of physiology as a *medical* science; as a science to which the student, and still more the practitioner of medicine, may confidently turn for the applications of those fundamental principles which constitute, in the long run, the only sure guides to success in the treatment of disease, and through which alone is the art of medicine to be advanced to the condition of an exact science.

Ever since the days of Harvey and of Haller, physiology has been making giant strides toward perfection. Nevertheless, strange as it may seem, the profession is still divided in opinion as to its utility and applicability to practical medicine. "When the cure of an order of diseases follows constantly the employment of a medication," says Renouard in his *Histoire de la Médecine*, "we are led to regard this medication as the cause of the cure which follows its use; but it is impossible for us to perceive the physiological reason of this result, and it is consequently useless to seek it. * * * The physiologist must limit himself to the description of the normal phenomena of the living economy—the pathologist to the abnormal ones." Contrast with this passage the subjoined language of another transatlantic author, who had in him the spirit of truth, when he wrote "that in no way is both the science and the art of healing so likely to be improved as by the association in its literature, and through that in the minds of its practitioners, of pathology with physiology rather than with morbid anatomy; that juster theoretical views are elicited by looking upon disease as part of the phenomena of life than as the producer of appearances seen after death; and that patients are more likely to be cured by one, whether original observer or reader, who is considering even imperfectly the vital actions exhibited by them, than if he knew exactly what would be the consequences of the disease in the corpse." And again: "It tends to false views of nature's works, and bad practice in our profession, to suppose physiology and pathology different sciences. It is a fatal error to do or to say anything which can lead to the idea, that in health one set of laws are exhibited by our bodies, and in disease another set; or, what is still worse, that these laws are in opposition the one against the other."* So, also, Bernard writes: "We are not authorized to regard physiology and pathology as two distinct domains, in which occur phenomena essentially different in character."† The same idea is expressed by Professor Bennett in other words. "Vital action, as manifested in the ultimate fibre of a muscle," says he, "may be con-

---

* Digestion and its Derangements, by T. K. Chambers, M.D.; New York, 1856, pp. 14, 223.

† Leçons de Physiologie Expérimentale; Paris, 1855, t. i. p. 24.

sidered histological, as appertaining to elementary structure; vital action, as exhibited in a group of muscles, for the purposes of speech, deglutition, respiration, locomotion, and so on, may be called physiological, as belonging to the functions of organs; and vital action in muscle, as shown in perversion of those functions during spasm, convulsion, or paralysis, is more properly called pathological. It must be evident, however, that these distinctions are altogether arbitrary, and that the three subjects constitute one study. Thus, our knowledge of muscular action is derived from an acquaintance with its ultimate structure and the contraction of its individual fibres, and from an observation of its diseased conditions. Where health terminates and disease begins, has never yet been settled; nay, more, what is health to one man would be disease in another, just as the degree of strength, which is natural to a delicate frame, would be considered weakness in a strong one. Histology, physiology, and pathology, then, are closely allied, and are only different divisions of the same subject; the facts of one are available for the study of all; and any theory deduced from this branch cannot be correct unless in accordance with the data furnished by the others."

Normal and abnormal processes are, indeed, wonderfully interwoven in the life of man. The organic harmony which we call health contains in itself the seeds of discord. In obedience to an unalterable law of the economy, these seeds sooner or later begin to germinate. Their germination and development are the avenues to death. Rightly considered, health is but a relative term. It not only expresses ease, but implies disease; so that the natural history of disease, which is pathology, is in truth a part of the natural history of ease or health, which is physiology. Whether by diminution or exaltation, pleasure, the sign of health, may be quickly converted into pain, the symbol of disease. As a comprehensive study of pleasure involves the investigation of pain, or as a complete knowledge of perfection necessitates an acquaintance with imperfection, so the laws of health must, to a great extent be elucidated by those of disease. Indeed, if "health is only the beginning of a just consideration of the human being," then are disease and death the end of that consideration; and this end, coming around circularly, touches at innumerable points upon the beginning.

The budding health of the infant, measured by a pulse of 120 beats, and a respiration of 30 movements in the minute, would be disease in the adult. So the health of the adult is not exactly health in the old. Hence, we speak of an infantile, a juvenile, an adult, and a senile physiology; while the pathologist describes for each of these periods a characteristic semeiology. In almost all hearty persons, temporary alterations of structure and function, of too slight a character to attract serious attention, are more or less constantly occurring. Between these slight alterations, and those extensive and persistent changes of structure and function, which are hopelessly fatal, the shades of transition are numerous. Some men are born with organs so well attuned to each other and to surrounding conditions, that they fill up the measure of threescore years and ten without disease, and then, decaying, slip away gently from the scene of their daily hopes and fears, in obedience to the inexorable law which assigns to the human organism a definite time to live. In others, again, and they are the majority, some one organ, from an inherent and hereditary inability to do its work properly, tends to decay sooner than its associates. Thus the sick organ precipitates that dissolution which we all feel must come at last.

All the details of the *ars medendi* tend to the conclusion, that pathology is simply an "erring physiology." Both are essentially processes, and not entities; processes, moreover, in which the various constituent acts or movements pass into and blend with each other so imperceptibly that they cannot be separated. Both may strictly be regarded as the manifestation or objective indication of the existence and activity of the life-principle. Both manifest a remarkable periodicity of action. The study of both has been pursued in accordance with the anatomical method, and with equally unsatisfactory results. The two sciences are, in truth, mutually elucidating parts of one continuous whole. The physiology that ignores pathology, or the pathology that repudiates physiology, is an incomplete and thriftless study, alike without principles and without beneficial results. If the slowly and laboriously accumulated history of medical doctrines teaches us anything, it is, that in the various epochs of that history the ruling pathological idea has been modeled after and based upon the prevalent physiological doctrine of the time. When the iatro-chemists of the seventeenth century maintained "that the operations of the living body are all guided by chemical actions, of which one of the most important and the most universal is fermentation, the states of health and disease were supposed to be ultimately referrible to certain fermentations, which took place in the blood or other fluids."* And so, toward the close of the same century, when the "body was regarded," by the iatro-mathematicians, "simply as a machine composed of a certain system of tubes, calculations were made of their diameter, of the friction of the fluids in passing along them, of the size of the particles and the amount of retardation arising from friction and other mechanical causes; the doctrines of derivation, revulsion, lentor, obstruction, and resolution, with others of an analogous kind, all founded upon mechanical principles,

* Bostock's History of Medicine.

were the almost universal language of both physicians and physiologists." And when, at a later period, the chemists and physicists were succeeded by the vitalists, who ascribed all the healthy operations of the body to a superintending *anima*, or unconscious soul, the physicians stoutly held that upon this necessary and unintelligent agent depended the prevention and repair of injuries and the removal of morbid conditions. The apparently modern, but in reality ancient physiological doctrine of dynamical or vital agency, deeply underlies and gives color to the medical science of the present day.

Physicians are not yet agreed whether the study of normal should precede or succeed the study of abnormal phenomena. And yet this important question must be settled before clinical medicine can attain the status of a science, inasmuch as the fundamental theory of the latter is to be determined by the result of the inquiry. The laws of pathology have not been, and, in all probability, cannot be, generalized from morbid phenomena alone. The rapidity with which groups of phenomena are generalized and reduced to a system is in direct proportion to their simplicity and freedom from perturbing influences. Hence, physical science is older and far more exact than organic science. So human physiology antedates human pathology; the phenomena of the former being simpler, and varying within more narrow limits than those of the latter. Some there are, who, like Coleridge, hold that the laws of disease are to be elucidated wholly from the phenomena of disease. On the other hand, Geoffroy St. Hilaire,* Bichat,† Ducrotay de Blainville,‡ Comte,§ Esquirol,‖ Robin and Verdeil,¶ Georget,** Feuchtersleben,†† Cullen,‡‡ Bowman,§§ Brodie,‖‖ Lawrence,¶¶ Simon,*** and many others, with a more philosophical conception of the relations of physiology to pathology, consider

---

* Hist. des Anomalies de l'Organization; Bruxelles, 1837, t. ii. pp. 9, 10, 127.

† Anatomie Génerale; Paris, 1821, t. i. p. 20.

‡ Physiologie Comparée; Paris, 1833, t. i. p. 20.

§ Cours de Philosophie Positive; Paris, 1830–1842, t. iii. pp 334, 335.

‖ Des Maladies Mentales; Paris, 1838, t. i. p. 111.

¶ Traité de Chimie Anatomique; Paris, 1853, t. i. p. 68.

** De la Folie; Paris, 1820, pp. 2, 391, 392.

†† Principles of Medical Psychology, Sydenham Society's edition; London, 1847, p. 200.

‡‡ First Lines of the Practice of Physic; Brookfield, 1807, vol. i., Preface and Introduction, pp. 23, 24.

§§ Encyclopedia of the Medical Sciences; London, 1847, p. 824.

‖‖ Lectures on Pathology and Surgery; London, 1846, p. 3.

¶¶ Lectures on Comparative Anatomy, Physiology, Zoology and the Natural History of Man; Bohn's edition, 1848, p. 45.

*** General Pathology; Philadelphia, 1852, pp. 12, 13.

that the latter should be studied chiefly as a deduction from the former, and not as an inductive science *per se.*

It is in consequence of entertaining such views of the relations of physiology to pathology, that we have been led to compare together the *Treatise* of Professor Dalton and the *Outlines* of Professor Bennett. Dr. Dalton is the normal or scientific, Dr. Bennett the medical or pathologic physiologist. The former treats his subject as a *pure* science; the latter exhibits it to us as an *applied* science. The object of Dr. Dalton is, as he indeed informs us in his preface, "more particularly to present, at the same time with the conclusions which physiologists have been led to adopt on any particular subject, the experimental basis upon which those conclusions are founded; and he has endeavored, so far as possible, to establish or corroborate them by original investigation, or by a repetition of the labors of others." The leading desire of Dr. Bennett has been, as we also learn from his preface, "to connect physiology with the scientific practice of the medical art." Dr. Dalton, the experimentalist, restricts himself to the field of healthy physiology, intent on verifying its facts and enlarging their number; Dr. Bennett, the practitioner of medicine, takes the established facts of physiology, and endeavors to show us how to use them in elucidating the obscure points of pathology and therapeutics. The *Treatise* of the former finds its proper place among the ordinary physiological text-books of the day; the *Outlines* of the latter may be regarded as the crude and imperfect exponent of a new school—the school of *applied physiology*—a school whose growing importance is acknowledged by all medical thinkers, whose method is peculiar to itself, and whose literature lies scattered in various physiological works, in special monographs on physiology, pathology, and therapeutics, in medical journals, in hospital reports, in treatises on hygiene, in the transactions of biological societies, etc. The *disjecta membra* of this literature are yet to be collected, arranged, and condensed into a scientific system—the true Institutiones Medicinæ. Hopeful attempts at the application of physiology to the explanation and treatment of disease, are to be found in the work of Chambers on *Digestion and its Derangements,* in that of Radcliffe on Epilepsy, that of Brinton on Diseases of the Stomach, and many others which we might mention. The most recent and philosophical effort in this direction is contained in the Lectures of Brown-Séquard, delivered at the Royal College of Surgeons of England, in May, 1858, and published in the *London Lancet* for that year.

A cursory examination of the standard physiological works of the day is sufficient to show that attempts have been made both to investigate and to teach physiology in accordance with systems fundamentally different from each other. Of these various systems only four occupy a prominent place in the history of medicine. These are the Circumstantial or strictly Phenomenal, the Historical or Progressive, the Anatomical or Organic, and the Physiological or purely Functional. The first two are chiefly methods of exposition or education; the last two provide for the gradual development and advancement of the science. To these may be added a fifth, which may be called Clinical Physiology, or Physiology in its application to Clinical Medicine, and which, though yet in its infancy, is destined to supersede all the others in our medical schools.

We proceed to examine them in detail. An opinion has long prevailed that the function of an organ could be readily inferred from its anatomy. This opinion has descended to us stamped with the seal of ancient authority. Galen long ago expressed it in the very title of his work, *De Usu Partium*, which title Lawrence considers to be well chosen. Long anterior to Galen, however, Democritus, of Abdera, who is said to have dissected many animals, investigated the source and passages of the bile with a view to determine the cause of madness. In his *First Anatomical Disquisition on the Circulation of the Blood*, addressed to Riolan, of Paris, in 1649, Harvey assures us that "the dissection of healthy and well-constituted bodies contributes essentially to the advancement of philosophy and sound physiology." In Dr. Ent's "Epistle Dedicatory," prefixed to Harvey's *Anatomical Exercise on the Generation of Animals*, the latter is represented as saying: "No one indeed has ever rightly ascertained the use or function of a part who has not examined its structure, situation, connections by means of vessels, and other accidents, in various animals, and carefully weighed and balanced all that he has seen." In another place, when expounding the method to be pursued in studying generation, he accuses Fabricius of "quitting the evidences of sense that rests on anatomy and seeking refuge in reasonings upon mechanical principles."

Haller, the father (as he has been called) of modern physiology, was so filled with the importance of anatomy to physiology that he deliberately spent thirty years of his life in anatomical pursuits, preparatory to the composition of his justly celebrated *Elementa Physiologiæ Corporis Humani*. In that work he calls physiology *anatomia animata;* and he advises us concerning it in these explicit words: "Et primum, cognoscenda est fabrica corporis humani, cujus penè infinitæ partes sunt. Qui physiologiam ab anatome avellere studuerunt, ii certè mihi videntur, cum mathematicis posse comparari qui machinæ alicujus vires et functiones calculo exprimere suscipiunt, cujus neque rotas cognitas habent, neque tympana, neque mensuras, neque materium." It was not, however, to special or descriptive, so much as to comparative anatomy, that Haller attached such weight. While he strongly recommends that the figure, size, and situation of the organs should be studied in the human body, he also observes that we must become acquainted with these points in the bodies of various animals if we would acquire a thorough knowledge of the uses and actions of the organs.* Thus he dissected the liver in man, quadrupeds, birds, fishes, and reptiles, and noticed that this organ in some animals was not associated with a gall-bladder. He next observed that every animal possessing a gall-bladder was also supplied with a liver. These facts taught him to regard the former organ as a mere appendage of the latter, and not as the true secernent of the bilious fluid found in both these parts. Thus he concluded that the function or use of the liver was to secrete the bile employed in digestion; and in this conclusion he was confirmed by discovering, furthermore, a channel of communication between the liver and gall-bladder by which fluid secreted in the former could readily find its way into the hollow reservoir of the latter. In like manner comparative anatomy teaches us that the essential or fundamental part of the acoustic apparatus is the vestibule—a sac upon which the auditory nerve is distributed. In the cephalopod and gasteropod mollusks, and in crustacea, this sac constitutes the entire organ of hearing. As we ascend through the animal series to man, the semicircular canals, the cochlea, the tympanum and its contents are successively added in accordance with the increased perceptive powers of the animals.

Despite the labors of Daubenton, Camper, Pallas, Harvey, Haller, Hunter, Blumenbach, Cuvier, and others, however, the capability of anatomy to unravel the complex phenomena of life has, at length, in our own day, come to be seriously questioned. Its manifest inability to answer satisfactorily the numerous and difficult questions of the physiologist has led the latter to seek for the explanation of these questions in another channel. We allude to vivisection, an important branch of experimental physiology. The celebrated Magendie, recently (1855) summoned by death from the field of his zealous pursuits, may be regarded as having fairly inaugurated this method of investigation. With him, however, it was at first an anomalous art, which the world, misunderstanding its object, regarded as more cruel than useful, more curious and puzzling than certain and instructive. In the hands of Claude Bernard, Brown-Séquard, and others of the French school; of Bidder and Schmidt, of the Derpt laboratory in Germany; and of Matteucci, in Italy,—it is slowly and surely assuming the character of a science. Half a century ago, Le

* In the Preface to his *Elements*, he says: "Quotidie experior, de plerarumque partium corporis animati functione non posse sincerum judicium ferri, nisi ejusdem partis fabrica et in homine, et in variis quadrupedibus, et in avibus, et in piscibus, sæpe etiam et in insectis inotuerit."

Gallois, in the Introduction to his *Experiments on the Principle of Life*, deemed it necessary "to exculpate the physiologists who make experiments upon living animals, from the reproaches of cruelty so frequently uttered against them." In England and our own country it is still thought necessary to apologize for the practice of vivisection.

Bernard considers anatomy as the *point d'arrivée* rather than the *point de départ* of physiological research. He reminds us of the ignorance of Sylvius, Varolius, and others, concerning the functions of the brain, notwithstanding their very minute and careful dissections of that organ. Meckel dissected the fifth pair of nerves, discovered its ganglia, and described its anastomoses without suspecting its functions. Microscopic anatomy appears in this respect to be nearly as deficient in results as both descriptive and comparative anatomy. By means of the compound microscope the human fabric has been unraveled, so to speak, and reduced to its ultimate constituents, the simple fibre and the organic cell, and yet, notwithstanding the searching structural analysis to which that fabric has been subjected, but little light has been cast upon the phenomena of living organized matter. "We have the most perfect anatomical knowledge of the spleen, thymus, and thyroid glands; but their offices in the animal economy are wholly unknown." Nearly forty years have elapsed since these words were pronounced by Lawrence before the Royal College of Surgeons.* During this time the accurate microscopic investigations of Müller, Giesker, Schwager-Bardeleben, Wagener, Panagiotides, Ecker, Lucæ, Haugsted, Rokitansky, Kölliker, R. Wagner, Landis, Gerlach, Sanders, Funke, Cooper, Simon, Gray, and others, have made us thoroughly acquainted with the minute structure of these organs. Nevertheless, in the spring of 1855, we find Bernard addressing his class in the following language: "Ne connaît-on pas aujourd'hui très-exactement l'anatomie microscopique de la rate, du corps thyroïde, des capsules surrenales? Connaît-on pour cela leurs fonctions? Non, et on ne les saura jamais que par l'expérimentation." In fact, anatomy acquaints us with the size, form, and structure of an organ. It thus prepares us for the study of the function of that organ; but does not, nor can it, of itself, reveal the function. To attain this a higher and more powerful instrument of research is necessary. The organ must be examined while acting, while performing its allotted part. The liver secreting bile or carrying on its glycogenic function, or manufacturing hepatine, if Pavy be correct, is a very different organ from the cadaveric liver bereft of that power.

The anatomical method starts with the following proposition: Given an organ, to determine its function. Such a proposition presupposes that

* Lectures, etc. p. 44.

the function is entirely located in a certain part. This, however, is not so. The functions are not objects tangible and well defined; neither are they isolated acts. They constitute rather a connected series of events, never constant, but ever fluctuating under the influence of objective as well as subjective conditions, like the ebb and flow of the unresting ocean. "We should always," says Beraud, "figure to ourselves the different acts embraced in the domain of physiology as dependent upon each other, occurring *simultaneously* and not *successively*." So close is the connection between these acts that any derangement of one is sooner or later accompanied with disturbance of the others; while in its turn each one is dependent for its regularity on all the others. The anatomist may dissect out and study separately each of the organs; but the physiologist cannot so isolate and examine the functions. A segregated function is an impossibility. Life is the sum-total of the functions; and this sum-total is a unit which cannot be decomposed and analyzed by the physiologist, as the chemist decomposes and analyzes a mineral. Any decided attempt at such decomposition necessarily involves the destruction of the analyte or subject to be analyzed. We may study most minutely the heart, or the lungs, or the digestive canal, on the dead subject, and be but little the wiser as to their functions. To acquire a knowledge of the latter we must patiently and laboriously watch the play of the former in the living subject. "An organ does not live by itself," very justly writes Bernard. * * * * "Only the organism, as a whole, lives and acts. If we study separately and in succession all the parts of any mechanism whatever, we can obtain no idea of its action. So in pursuing physiology anatomically, we may take the organism to pieces, but we cannot on that account grasp the idea of it as a whole. This idea becomes visible only when the organs are in motion." Although comparative anatomy, in the hands of a Haller, might furnish us with some idea of the secretory function of the liver, yet it is very evident that neither the descriptive, comparative, nor microscopic anatomy of the day could determine that this same liver was also a sugar-elaborating apparatus. Bernard expressly assures us that this important discovery, for which we are indebted to him, was accidentally made during an attempt to study a certain organic phenomenon—the transmutation of sugar in the animal economy—independently of all anatomical aids.

Why is it that anatomy has so signally failed to elucidate the problems of physiology?

From long observation, assisted occasionally by experiment, we learn to regard the forms and properties of bodies as the natural accompaniments of each other. A certain form becomes for us the symbol or measure of certain properties. Moreover, we learn to anticipate a change of properties as the necessary result of a change of form. Now, between the forms and properties of bodies, both organic and inorganic, there is undoubtedly a constant and determined relation. The exact nature of this relation,—whether it be one of cause and effect, which, notwithstanding the affirmative decision of Reil, Valentin, and other bold thinkers of Germany, there is strong reason to doubt, or one of simple association brought about by the action of the formative principle displaying itself through different media,—it is unnecessary to discuss in this place. We have been at some pains to collect and tabulate in another place* the facts bearing upon this question as it applies to the inorganic world.

The human mind, ever attempting to explain the unknown by the known, unconsciously judges of the properties of a body presented to it for the first time, by the knowledge which it possesses of the qualities of another body of analogous form and composition. The opinion arrived at is subsequently corrected by additional observation and experiment. The hasty and injudicious application of this analogical principle or method to the sciences of anatomy and physiology has given rise to much confusion. Reasoning applicable to the physical and chemical phenomena of the inorganic world has been applied without correction to the organic, and error has been the result. The early anatomists, as is well known, named various parts of the body in consequence of their real or fancied resemblance to objects with which they were familiar. Misled in part by these form-resemblances, which are in most cases simple coincidences, and overlooking the fact that the objects compared are not strictly analogous, but merely similar in one respect, physiologists have been led into a fundamental error by attempting to deduce the functions of certain organs from the uses or actions of bodies to which these organs bore some outward resemblance. Forgetting, moreover, that analogy is not homology, they have even attempted, from these vague resemblances, to argue not only similarity, but even identity of action. We are told, for example, that from their form we judge the stomach and urinary bladder to be reservoirs; the blood-vessels, canals for the conveyance of fluids; the bones, levers, etc. At first sight there is some show of truth in this procedure. When we attempt, however, to determine the true nature of muscles and nerves, in the same way, the fallacious character of the method becomes very apparent in our utter want of success. The organs first mentioned play a more or less mechanical part in the body, and the idea of their uses has been deduced not so much from their form alone as from the resemblance of this form to certain mechanical implements employed in the arts, etc. The failure to divine the functions of muscles and

* Journal of the Academy of Natural Sciences of Philadelphia, vol. iii. part ii.

nerves is clearly due to the fact that these resemble in form and structure nothing with which we are acquainted. Herein, then, lies the explanation of the inherent difficulty of the anatomical method of investigating physiology. Even among inorganic bodies which are characterized by such a remarkable constancy of form and simplicity and homogeneity of constitution, the relations of form to composition and properties are as yet entirely too indefinite to be made the basis of any accurate method of study. Still more is this the case with the organic world, whose objects are so complex in structure and so variable in form and functions. From the simple animal cell, man, the head and front of the creation, is developed; in the simple vegetable cell the goodly oak takes its origin. Neither the most skillful chemist nor the most expert microscopist can point out any essential difference between these two cells. Physically and chemically they seem alike. Yet reason tells us that there is some difference, inappreciable though it may be. So long, therefore, as we are called upon to witness such different physiological results flowing from differences—whether of form, structure, or composition—so slight as to be beyond detection and demonstration, so long will we be prevented from using anatomical forms as the indications of functions. From identity of pro perties or functions in different bodies, we may justly argue homology of ultimate form and composition, and from similarity of properties we may argue analogy of form. But as our physical and chemical appliances, in most instances, prevent us from seizing ultimate and truly primitive forms, we cannot so surely arrive at a knowledge of functions from an acquaintance with forms. The science of morphology, both organic and inorganic, must be far more advanced than it is at present.

We have every reason to suppose that dead tissue differs molecularly from living tissue, even though we be not able to demonstrate this difference. The functions of which the physiologist treats are the properties, so to speak, of the living and not the dead tissues. Bernard has happily seized and forcibly dwelt upon this idea. "In physiology," says he, "the vital properties of living matter should be verified upon the living tissues; on no account should they be deduced from the conformation of the dead body."

In the *Introduction* to Professor Dalton's work, we find the following language, which is in general accordance with the foregoing views :—

"There is only one means by which physiology can be studied; that is, the observation of nature. Its phenomena cannot be reasoned out by themselves, nor inferred, by logical sequence, from any original principles, nor from any other set of phenomena whatever." We cannot "infer the truths of physiology from those of anatomy, nor the truths of one part of physiology from those of another part; but all must be ascertained directly and separately by observation. For although one department of natural science is almost always a necessary preliminary to the study of another, yet the facts of the latter *can never be in the least degree inferred from those of the former, but must be studied by themselves.*"

The physiological or functional method of investigating physiology proposes to start with the particular organic action or phenomenon to be studied, and by observation and experiment to track it through all its modifications and transmutations in the economy, until we are able to localize it anatomically. Instead of starting with the organ and terminating with the function, attention is first directed to the function, and last of all to its anatomical seat or centre. Following this method, the physiologist attempts to study the living being exactly as he finds him surrounded by constantly varying circumstances, and exposed to climatic conditions, which are, in truth, the external conditions of vitality. The reciprocal actions incessantly going on between these circumstances and conditions on the one hand, and the phenomena of the living organism on the other, constitute a deeply interesting chapter in physiological science, and one wholly beyond the capabilities of anatomy to explain. Bernard is the most determined representative of this method. He thinks, in fact, that experiment upon the living body ought to take the lead in all our studies of life-phenomena, and that the true value of anatomy lies in the *a posteriori* explanation of the phenomena discovered by physiological experimentation—a knowledge of the structure, form, and relations of organs, enabling us to account for functional peculiarities rather than for the function itself.

It will thus be seen that the experimental method so strongly advocated by Magendie, Bernard, and others, investigates and teaches simultaneously. Explorations and discoveries in new and untrodden fields are carried on before the student, who is himself stimulated to become an investigator by thus learning the manner in which physiology advances to new triumphs. One of the chief defects of this method, however, lies in its incompleteness as a means of instruction; facts and doctrines which are old and well established not being dwelt upon at all, or only incidentally. Novel questions claim profound attention, and the interests of the science are cared for at the expense of the wants of the student. In the College of France, to which Bernard is attached, attempts are constantly made, through new investigations and discoveries, to fill up the scientific lacunæ overlooked in a more systematic course. To use the language of Bernard himself, employed at the opening of the *cours du semestre d'hiver*, 1854–55, "in preference to established questions, the most difficult and obscure problems are attacked in the presence of an audience already, by their previous studies, prepared for discussion." With the *Faculté de*

*Médecine*, on the contrary, the system of exposition is at once dogmatic and synthetical; undisputed facts are presented to the exclusion of others, and by the use of theories and hypotheses, oftentimes more ingenious than ingenuous, the science is made to assume the appearance of a unit instead of the fragmentary and incomplete study, which it really is. The faculty professor is the descriptive geographer, who tells us of lands explored and known; the experimental biologist is the bold navigator, who forsakes the beaten waters, and turns the prow of his ship toward new and strange lands, ever ready to alter his course as circumstances may arise requiring it. The experimentalist teaches by example, the faculty professor by authority. The one investigates, the other narrates. The one relies upon the knife and the retort, the other upon records and history. As a conquering army marching through a hostile country attacks and overcomes town after town, so the experimental physiologist discovers and elucidates the various acts or functions of the organic body, abandoning each in turn, as soon as he has mastered its details, and pushing on to new conquests. And as the historian, in after times, comes to write out in detail, and comment upon the acts of the victorious army, so the faculty professor, from time to time, records the new discoveries of the vivisector, criticises and compares them with the old, and assigns them a value and a place in the great standard volume of physiology, which is rapidly attaining such unwieldy proportions. Another defect of the experimental method, which must not be overlooked, is the extreme difficulty of applying it successfully to the elucidation of many of the phenomena which we witness in living beings. Experimental biology differs in several important particulars from experimental physics and chemistry. The physicist and the experimental chemist have it in their power greatly to simplify their investigations, by withdrawing the problem to be solved, or the body to be examined, from all disturbing conditions which might in any manner conflict with the truthfulness of the results obtained. In other words, they can conditionate their analyses, and prescribe limits to their problems, without in the least affecting the essential nature of either of these. Not so, however, the biologist; any attempt on his part at such isolation alters at once the fundamental character of the phenomenon or function to be studied. Each and every organic act must be investigated just as we find it in nature, encumbered with a greater or less number of associated elements, inasmuch as the elements are the essential and modifying accompaniments of the phenomenon. In fact, the latter is what it is on account of the former. The functions of the living organism act and react upon each other without let and without stay. In experimenting upon the higher animals, the most skillful vivisector is very apt to disturb the function which he desires to study. The influence of this function upon the others is impaired, and the latter, in their turn, react upon the former in an irregular and unaccustomed manner. Thus arises a series of perturbations in the economy, which it is difficult to eliminate or make allowance for, and which, if not eliminated, seriously obscures and embarrasses the whole investigation. As much care is required, therefore, in the preliminary institution of an experiment as in its subsequent execution. Hence, the wary philosopher, who comes after the experimental pioneer, to obtain and use his facts for the lofty purposes of generalization, always anxiously inquires how the experiment was performed, and what were its attendant circumstances. Without this necessary information, "science is justified," as De Blainville long ago said, "in refusing to admit the experiment, especially if its results contradict those already established by analogy." Against vivisection may also be urged the impossibility of performing upon man many of the experiments to which the lower animals have been subjected; and the consequent liability to error, which must always, in a greater or less degree, accompany any attempt at explaining the healthy functions of one species or genus of animals, by the results of experiments performed upon those of another species or genus. However, of the hygienic, therapeutic, and vivisectal *modes d'experimentation*—employed separately and in equally skillful hands—the last is undoubtedly the most productive of extensive and important results. Its value is inversely as the difficulty of execution, and directly as the simplicity and independence of the function and the fewness of its associated elements.

The methods of teaching physiology next claim our attention.

The lecturer who follows the circumstantial method, after some preliminary remarks upon the character and objects of his science, the difference between organic and inorganic bodies, the nature and origin of organized matter, the chemical and structural composition of the human body, and its vital and physical properties, proceeds to take up the various functions seriatim, and to narrate, in a certain arbitrary or conventional manner, the numerous facts which, from time to time, have been placed upon record by different observers and experimenters. Of these observers and experimenters little or nothing is said, nor is allusion often made to the objects for which these labors were undertaken, or the animus which guided their performance. But oftentimes the value of a series of experiments and observations is to be determined partly by a knowledge of the objects for, and the manner in, which they were performed, and partly by the manual dexterity and theoretical bias of the performer. It is in consequence of a neglect of these points that so many "false facts," or errors in the disguise of truth, have crept into the temple of our science, only to mystify and perplex those who serve at its altars. Taught in accordance with the method under consideration, physiology is divested of all real philosophy, and presented to the student as a series of isolated facts, more or less true,—a lifeless and motionless tableau, as it were, of details, rather than of principles. Like the positive philosophy of August Comte, it is strictly phenomenal; and like that philosophy, it is also more positive than philosophical, since it ignores the great principle of causation as displayed in the laws of formation and development, and in the fundamental dogma or truth of physiology—the functional unity of organisms in time and space. The circumstantial method endeavors to give a thorough and settled view of the science, by skillfully dwelling upon its well-established points, and passing by in silence, or with a few dogmatic and oracular words, the numerous questions still mooted and unsettled. In this manner the science is made to assume an appearance of maturity and completeness more specious than real. The student is thus deceived and led astray at the very threshold of his studies, so that when he comes to make some practical application of his physiological knowledge, being without principles, it fails him in the hour of his greatest need, turning like fairy money into leaves and dust. With the industrious lover of truth, earnest investigation speedily follows this unlooked-for result, and very soon the student tears aside the veil and exposes the boasted science in all its naked uncertainty. Before him, in a sandy waste of doubt and perplexity, a few green and fertile oases appear, and he is ready to exclaim that our knowledge of physiology, to use the language of Bassanio in the play, is "as two grains of wheat hid in two bushels of chaff; you shall seek all day ere you find them; and, when you have them, they are not worth the search."*

It is contended, we are well aware, that the student during his collegiate career is in the position of a receiver and storer up of knowledge, and not an examiner, and that it is his duty to learn and remember as many of the undoubted facts of physiology as he can. Mental digestion and criticism of these are to come afterwards at his leisure. This we are inclined to consider as radically wrong. It appears to us as improper to crowd suddenly upon the brain a mass of information, as it is to fill the stomach at once with a superabundance of food. In the one case a mental apoplexy, and in the other a fit of indigestion are the results. The student should rather be taught to think and digest as he goes along. He should be made acquainted with the doubtful and disputed as well as the established. It is important for the traveler to know the dangers as well as the beauties of the road he is to traverse. Forewarned, he may escape the one, while enjoying the other. In like manner, the really judicious

* Merchant of Venice, Act I. Scene 1.

father, in counseling his son, points out to him the evil that is in the world as well as the good. So the teacher of physiology should take good care to demonstrate all the deficiencies and shortcomings of his science as well as its great and undeniable facts and principles, that the student may thus acquire a truthful idea of the value of the science, and know exactly what dependence may be placed upon it in the performance of his duties as a practical physician. Ere he essays the battle, the warrior looks well to his trusty sword; he examines its handle, the weight and elasticity of its blade, the sharpness of its point, the keenness of its edge, and the like. He notes well its weak points, and estimates its capabilities for the fight. Physiology is the sword with which the physician encounters disease. Its true value lies in the knowledge which the practitioner has acquired of its nature and its powers. Lacking this knowledge, it is but a useless weapon of glass within his hand.

By the historical method, physiology is treated as a living and progressive spectacle, instead of a motionless and meaningless tableau. The knowledge which we possess, at the present day, of each of the various functional acts of the economy, is presented to the student in the chronological order,—the order, in fact, in which the various discoveries appertaining to it were made. The student is thus enabled to traverse the whole ground for himself, step by step, and to observe how truth after truth was established, and how one great result sooner or later led to another. This is the method adopted by Milne Edwards, in his valuable course of lectures on "Physiology and the Comparative Anatomy of Man and Animals," delivered *à la Faculté des Sciences de Paris*, and now in course of publication. In the first volume, he says: "In beholding the manner in which the science is constituted, and has grown little by little, we the better seize its spirit and its method; we become acquainted with the men as well as with the things, and we are inspired with a just respect for the labors of the investigators of nature, even while the fruits of their labor have not yet become apparent; for in this study we recognize many examples of facts, which, after remaining for a long time barren and neglected, have suddenly become the germ of a great discovery, when the moment arrived to comprehend their bearing, and a man of genius appeared to attach to them his seal." A moment's reflection will show that to present the facts of physiology in their chronological order, is also, in great part, to present them in the logical order. For the acquired facts of one period in the march of science are very frequently the natural and essential precursors of those which appear at a later epoch; so that the order in which the facts of physiology were discovered often expresses truly their natural relation.

The progressive or historical method is undoubtedly the best calculated to instruct the student, and give him a comprehensive and profound view of physiological science. Nevertheless, it cannot, at present, be thoroughly adopted in our schools. Want of time alone forbids its introduction. Edwards, its great advocate, who carries it more extensively into practice than any other physiologist, occupies the first volume of his published lectures, a royal octavo of 535 pages, with the consideration of the blood and respiration alone. The latter is commenced in the first volume, and continued throughout the whole of the second, which contains 655 pages. The third volume, comprised in 614 pages, is devoted entirely to the circulation and the circulatory apparatus. But Edwards assures us that the number of lectures composing the six months' course on physiology and comparative anatomy does not permit him to enlarge equally upon the history of all the functions.

In Europe, where medicine is divided into a greater number of branches, which are taught as specialties, in a more elaborate manner, and perhaps in a more philosophical, though less practical spirit than in our own country, and where a chair is especially devoted to the exposition of pathology, both the historical and experimental methods may be and are pursued with greater advantage to the student, who, if he be diligent and make the best use of his time and opportunities, cannot fail to go forth well prepared for the active duties of his profession. In our own country, where medical science is taught from seven chairs only, instead of ten or a dozen, as the exigencies of the times and the science will sooner or later demand, and where etiology, pathology, hygiene, and therapeutics, under the comprehensive title of the Theory and Practice of Medicine, are taught in about one hundred lectures, by one professor, assisted in a desultory or incidental manner by the other professors, especially those of physiology and materia medica,—neither of these systems of teaching is applicable. Some Bernard might occupy one course with a valuable series of experimental lectures on a particular point connected with the digestive functions; and in the next course he might in the same way direct attention to some other topic. Those students who graduated at the end of this, their second course, might be well grounded in the physiology of digestion, but would know literally nothing of the rest of the science, and especially would this be the case, if the lecturer, as is the custom with us, were to repeat the same course year after year. Obviously this method is not adapted to the genius of our system of medical teaching, if, indeed, we can ascribe genius to a system so cramped in time, and, therefore, in many instances, so uncertain in results. In our schools, the teacher of experimental physiology would be forced to one of two courses: either he must teach a very few subjects thoroughly, to the utter neglect of all others, or he must teach a great deal in a very hurried and inexact manner. We need scarcely say that hurried experiments, in connection with vital phenomena, are worse than no experiments at all. Every one knows that in a decidedly experimental lecture, the hour is pretty evenly divided between the enunciation of the facts, or principles, and their demonstration, which is but a practical repetition of these facts, undertaken with the view as much to enforce their remembrance as to prove their accuracy. Hence it is, that experiments occasionally performed to illustrate some particular topic are often worse than useless, in consequence of their inefficiency. Moreover, in the hands of men less expert, less enthusiastic, and less philosophical than Bernard, they are apt to degenerate into a species of scientific jugglery, whose chief object appears to be to attract and amuse, rather than to instruct a class. These remarks do not apply to chemical lectures, nor to the physiological laboratory. The experiments illustrating a chemical course are, for the most part, simple and easy of execution, and really useful in assisting the student to remember some, at least, of the numerous details which constitute the science. But with physiology the experiments are too complex, and require too much time to be undertaken anywhere with success, but in the laboratory.

The first three chapters of Dr. Dalton's work are devoted to the consideration of the proximate, or, as Chevreul called them, the immediate principles. Following Robin and Verdeil, Dr. Dalton divides these principles into three groups, viz.: 1st. Inorganic substances; 2d. Cystallizable substances of organic origin; 3d. Organic substances proper. Under the first head he treats briefly of water, chlorides of sodium and potassium, carbonates of lime, soda, and potassa, and phosphates of lime, magnesia, soda, and potassa. It will thus be seen that not more than one-half of the principles belonging to this group are here noticed by our author. He very justly defines a proximate principle to be "any substance, whether simple or compound, chemically speaking, which exists under its own form, in the animal solid or fluid, and which can be extracted by means which do not alter or destroy its chemical properties." The thoughtful student might ask why the term simple was introduced into this definition, seeing that no examples of simple proximate principles are alluded to. And if he followed the inquiry up, and discovered that oxygen, nitrogen, and carbonic acid, existing as they do "under their own form" in the blood, are fairly entitled to be regarded as proximate principles,—the two former of the simple class,—he would naturally and justly take exception to the statement made by Dr. Dalton, that all the proximate principles of the first or inorganic group are crystallizable. Hydrogen, carburetted and sulphuretted hydrogen, are also ranked among the immediate principles by Robin and Verdeil. But these substances not being found in any of the tissues or fluids, but only in the air-passages and alimentary canal, they cannot be considered as compositional. Under the head of proximate principles of the second class, sugar, starch, and fats are discussed in a clear and instructive manner. The proximate principles of the third class are described correctly, but with great brevity. The description of these substances, together with some remarks upon their origin and destruction; upon the general characters, chemical constitution, and hygroscopic properties of organic substances; and upon coagulation, catalysis, fermentation, and putrefaction, are all condensed into the short space of ten pages.

Dr. Bennett is still more brief in his treatment of these compositional principles. He divides them into four groups—the albuminous, fatty, pigmentary, and mineral—and presents them to the student in the most meagre and unsatisfactory outline.

In chapter five, Dr. Dalton exhibits to us a "bill of fare," which, although very substantial, is not altogether unexceptionable on the score of variety. He describes very accurately the composition and nutritive value of cow's milk, wheat flour, dried oatmeal, eggs, and meat, but says never a word about tea, coffee, or any other of the accessory or auxiliary aliments. The interesting and really valuable labors of Böcker, Falck, Bischoff, Boussingault, Moleschott, Masing, J. Lehmann and many others, are thus passed over in complete silence. And yet a familiar acquaintance with the results of these labors is essential to both the student and the physician—the two classes for whom the book before us was written. Without this knowledge the student cannot hope to obtain a thorough and comprehensive view of the great act of nutrition, involving as this act does that of disintegration or disassimilation, as well as of assimilation. Nor can the physician prescribe intelligently, that is to say, physiologically, for the dietetic management of the sick, without such information. In common with the best physiological authorities of the day, Dr. Dalton very properly objects to Liebig's division of aliments into combustible or heat-producing and nutritious or plastic. At the close of this chapter he comments very judiciously upon the nutritive value of different substances, the total quantity of food required by the adult, and the preparation of food by cooking.

In his observations upon aliment, Dr. Bennett reproduces chiefly the generalizations of Liebig relative to the chemical principles which enter into the constitution of the living being to be nourished; the mode in which these are combined to form tissues and organs; and the influence of the atmosphere and of bodily and mental exercise in determining the kind and quantity of food to be consumed.

Chapter six of Dr. Dalton's work treats of digestion—a function whose elucidation has severely exercised the experimental ingenuity of many eminent physiologists. The literature of this function is so extensive, the observations and experiments relating to it so numerous, varied, and conflicting, and the well-established points so few, that we have always approached the discussion of this subject, in our annual course of lectures on physiology, with not a little hesitation. It is, indeed, no easy task to condense the facts concerning digestion into a summary at once thorough, concise, accurate and reliable. Yet this, we are pleased to find, has been, to a very considerable extent, accomplished by Dr. Dalton.

The various changes which the food undergoes when brought in contact with the saliva, gastric juice, bile, pancreatic fluid, etc. are all detailed in a very satisfactory manner. In estimating the total quantity of saliva secreted in twenty-four hours, Dr. Dalton does not place much reliance upon the quantity formed during the intervals of mastication, but prefers to ascertain how much is really secreted during a meal over and above that which is produced at other times. He has found, by experiments performed for the purpose, "that wheaten bread gains during complete mastication fifty-five per cent. of its weight of saliva; and that fresh-cooked meat gains, under the same circumstances, forty-eight per cent. of its weight. He estimates that the daily allowance of these two substances, for a man in full health, is nineteen ounces of bread and sixteen ounces of meat. The quantity of saliva, then, required for the mastication of these two substances is, for the bread 4572 grains, and for the meat 3360 grains. If we now calculate the quantity secreted between meals as continuing for twenty-two hours at 556 grains per hour, we have :—

| | |
|---|---|
| Saliva required for mastication of bread . . . | 4572 grains; |
| Saliva required for mastication of meat . . | 3360 " |
| Saliva secreted in intervals of meals . . . | 12,232 " |
| Total quantity in twenty-four hours . . | 20,164 grains; |

or rather less than three pounds avoirdupois."

The true function of the saliva, according to Dr. Dalton, is a purely physical one. Its action, he maintains, is simply to moisten the food and facilitate its mastication, as well as to lubricate the triturated mass, and assist its passage down the œsophagus. Though he acknowledges that saliva is capable of converting starch into sugar, yet he denies that this takes place in the human economy. In his remarks upon the gastric juice, our author appears to favor the idea of Lehmann, Bernard, and others, that the acid of the gastric juice is the lactic, and not the hydrochloric, as maintained by Prout, Braconnot, Dunglison, Bidder, Schmidt, and others. From the subject of intestinal digestion, which is ably treated, Dr. Dalton passes very abruptly to that of absortion, without deigning to notice the process of fœcification or the act of defecation.

In chapter seven, the process of absorption is described in a brief, but very accurate and lucid manner. No account is given of the agents which propel the chyle and lymph onward in their respective vessels. Cutaneous absorption is not noticed, although in a physiologico-medical point of view highly interesting and important.

In chapter eight the bile is considered. Most of the facts contained in this chapter were originally contributed by our author to the *American Journal of Medical Sciences* for October, 1857, in an essay "On the Constitution and Physiology of the Bile." From his numerous and careful experiments, he arrives at the following conclusions: That the crystalline and resinous biliary matters are not the same in different species of animals, though they resemble each other in most of their chemical properties, and act in the same way with Pettenkofer's test, whether one or both of them be present; that in different kinds of bile they are to be distinguished from each other principally by their reaction with the salts of lead; that in human bile there is no crystallizable biliary substance, but only a resinous one; that Pettenkofer's test is the only available one for the biliary substances proper; that the bile in carnivorous animals passes into the intestine for at least twelve days after the last meal; that it is discharged into the intestine most abundantly immediately after feeding; during the remainder of the twenty-four hours its flow is about uniform, (sixteen grains of biliary matters per hour in a medium-sized dog,) except from about the eighteenth to the twenty-first hour, during which time it is much less; that when bile comes in contact with the gastric fluids, the organic matters of the latter are precipitated; but the biliary substances remain in solution; that these substances disappear during their passage through the intestine, so that they can no longer be recognized by Pettenkofer's test; and that they are, in all probability, reabsorbed into the blood; but if so, they first undergo in the intestine such changes, that they no longer give Pettenkofer's reaction with sugar and sulphuric acid.

The formation of sugar in the liver constitutes the subject-matter of chapter ninth. The glycogenic function of the liver, as is well known, was discovered and announced by Bernard in 1848. Dr. Dalton having repeated the experiments of this eminent physiologist, and satisfied himself of their correctness, strongly advocates his views. He says:—

"If a carnivorous animal, as, for example, a dog or a cat, be fed for several days exclusively upon meat, and then killed, the liver alone of all the internal organs is found to contain sugar among its other ingredients. * * * The tissues of the spleen, the kidneys, the lungs, the muscles, etc. give no indication of sugar. Every other organ in the body may be entirely destitute of sugar, but the liver always contains it in considerable quantity, provided the animal be healthy. Even the blood of the portal vein contains no saccharine element, and yet the tissue of the organ supplied by it shows an abundance of saccharine ingredients. * * * The sugar which is found in the liver after death is a normal ingredient of the hepatic tissue. It is not formed in other parts of the body, nor absorbed from the intestinal canal, but takes its origin in the liver itself; it is produced, as a new formation, by a secreting process in the tissue of the organ. * * * The formation of sugar in the liver is, therefore, a function composed of two distinct and successive processes, viz.: first, the formation in the hepatic tissue, of a glycogenic matter, having some resemblance to dextrine; and secondly, the conversion of this glycogenic matter into sugar, by a process of catalysis and transformation."

In discussing the above views, our author makes no mention whatever of the able experiments of Figuier, Sanson and Colin. The still more valuable experiments of Pavy were published too recently to be available to him. In January, 1855, Figuier read before the Academy of Sciences of Paris an excellent experimental paper on the origin of the sugar contained in the liver. In his paper he arrives at conclusions directly opposed to the views of Bernard. He contends that the "liver in man and in animals is not endowed with the power of forming sugar, and that all the glucose which it contains in its tissues comes from without, that is to say, from the nourishment." He demonstrates the presence of sugar in the blood generally as well as in the liver. He deprives blood of its fibrin, and mixes it with three times its weight of alcohol at 36° C. "After a few minutes the blood is completely coagulated into a clot of a beautiful red, by the simultaneous precipitation of the globules and of the albumen of the serum. It is then strained through a piece of cambric muslin and pressed, and the residue is washed with a little alcohol. The liquid, when filtered, passes through almost colorless, manifesting an alkaline reaction." Acetic acid is then added, and the whole evaporated upon a sand-bath. A small portion of albumen is thrown down, leaving a residue containing glucose in combination with certain mineral salts. The glucose is readily detected by Trommer's test, and by fermentation. Figuier, by this process, found in the liver of the rabbit one per cent. of glucose, and in the blood of the same animal fifty-seven per cent. In the blood of the ox he found forty-eight per cent., and in that of man fifty-eight per cent. From his experiments, he concludes that "the liver would contain, in equal weight, scarcely twice as much sugar as the blood contained in other parts of the body." He accounts for this difference by regarding the liver as the storehouse in which is concentrated or accumulated, preparatory to its delivery into the circulation, all the glucose formed during digestion. Sanson* found glycogenic matter in the portal blood of both the herbivora and carnivora,

* Brown-Séquard's Journal de la Physiologie, April, 1858.

and Colin detected it in the chyle of these animals. Sanson regards the glycogenic matter of Bernard as dextrine, a substance which he says is found in the blood and various structures of the body, as well as in the liver. This latter organ does not produce glycogene, but merely abstracts it from the blood, and transforms it into sugar with greater activity than any other part of the body. Still more recently Pavy* attempts to show that the so-called "glycogenic function" of the liver is a post-mortem phenomenon, sugar being formed in this organ after death by the fermentation of hepatine—"a material particularly belonging to the liver, and secreted by it from saccharine, amylaceous, and other principles contained in the blood circulating through its capillaries." Dr. Dalton treats this whole question in a purely physiological manner. Its medical relations are entirely neglected; and yet these relations are of the highest importance, for the rational treatment of diabetes mellitus, for example, must to a great extent be conducted in reference to such investigations which are calculated to throw light not only on the treatment, but also on the nature and prophylaxis of this disease.

Chapter ten, on the spleen, contains nothing new or specially interesting.

The next chapter treats of the blood, to the consideration of which fluid our author devotes 19 pages. Milne Edwards's account of it occupies 339 pages of the first volume of his elaborate work. The reader will, of course, understand from the title of his work, that Edwards does not confine himself to the description of human blood alone. In his description of the structure of both the red and white corpuscles, Dr. Dalton is in positive conflict with the opinions entertained upon this subject by the most eminent physiologists and microscopists. He says that each blood-globule consists of a mass of organized animal substances, perfectly or nearly homogeneous in appearace, and of the same color, consistency, and composition throughout. In no instance is there any distinction to be made out between an external cell-wall and "an internal cavity." He also denies the existence of a nucleus in the white corpuscle, regarding it as "merely an appearance produced by the coagulating and disintegrating action of acetic acid upon the substance of which it is composed." He maintains, furthermore, that the red and white globules are distinct and independent anatomical elements; that the latter are never transformed into the former, and that it is not at all probable "the red globules are produced or destroyed in any particular part

* Guy's Hospital Reports, October, 1858. See also the Pacific Medical and Surgical Journal, April, 1859.

of the body." Did our space permit, we might readily cite many authorities against these peculiar though not entirely novel views.

In chapter twelve, respiration is described in the same clear, concise and happy manner which characterizes other portions of the work. The renovation of the air in the pulmonary lobules and vesicles is accomplished, according to our author, by the inspiratory and expiratory movements of the chest, by the diffusive power of the gases which are interchanged, and by ciliary motion. The thoracic movements account very well for the introduction of fresh air into, and the discharge of foul air from, the trachea and larger bronchial tubes. Gaseous diffusion alone will not account for the transference of the fresh air from these tubes down into the pulmonary cells. Dr. Draper has shown that this transference, if conducted on that principle alone, would require a period greatly beyond the time occupied by one respiratory act, which is about three seconds and a half.* A force auxiliary to that of diffusion is therefore required to accomplish this transfer. This force Dr. Dalton finds in ciliary motion, which, he says, produces a double stream of air in each bronchial tube; "one current passing from within outward, along the walls of the tube, and a return current passing from without inward, along the central part of its cavity." We are inclined to think that ciliary action is scarcely sufficient to establish such regular and efficient currents. Kirkes and Paget believe that the continual vibration of the cilia may serve to prevent the adhesion of the air to the moist surface of the bronchial membrane. Todd and Bowman regard these cilia as agents of expectoration simply. Williams attempts to prove, and we think successfully, that these motive organules enact no part in the office of respiration, but subserve a merely mechanical purpose in the process of excretion.† We are inclined to think, with Dr. Draper, that physiologist of lofty and comprehensive views, that in the circular organic fibres of the bronchial tubes, we are to look for the agent which assists diffusion in bringing about an interchange of gases in the pulmonary cavity. The observations and experiments of Reisseisen, Meckel, Laennec, Williams, Longet, and Volkmann, show that the lungs are not passive organs, as Mayow thought, but in reality exhibit decided contractile power. During inspiration the entering column of air must put the muscular and elastic tissue of the bronchial tubes upon the stretch; during expiration force is generated by the active contraction of the muscular fibres, and the passive recoil of the elastic tissue. Dr. Radcliffe Hall‡ thinks that the force thus

* American Journal of Medical Sciences, April, 1852.

† Cyclopedia of Anatomy and Physiology, parts 45, 46; Organs of Respiration, by Dr. Thomas Williams.

‡ Transactions of the Provincial Medical Association, 1850.

engendered assists materially in the expulsion of air from the lungs. To this opinion it is objected that the contraction of the bronchial tubes during expiration, especially if it occurred at the commencement of the latter act, would tend to arrest rather than hasten outward the advancing aerial column. According to Dr. Draper, "the carbonic acid, vapor of water, and excess of nitrogen, if any, that have accumulated in the cells belonging to any given bronchial tree, are expelled therefrom by the muscular contraction of the circular organic fibres, and are delivered into the larger bronchial tubes, in which diffusion at once takes place with the air just introduced. As soon as the expiration is completed, relaxation of the muscular fibres occurs, and the passages and cells dilating, both through their own elasticity and the exhaustive effect arising from the simultaneous contraction of other bronchial trees, fresh air is drawn into them, the alternate expulsion and introduction being accomplished by muscular contraction and elasticity, the different bronchial trees coming into action at different periods of time, some being contracting while others are dilating." The passage of oxygen from the air-cells into the blood, and of carbonic acid from the blood into the pulmonary vesicles, is regarded by Dr. Dalton simply as a "double phenomenon of exhalation and absorption." It is an obscure and very complex process, in which a powerful condensing action is brought into play by the walls of the air-cells and the coats of the pulmonary capillaries. Dr. Bennett, unacquainted with the instructive experiments of Dr. Draper and the late Dr. J. K. Mitchell, still adopts the fallacious statements of Valentin concerning the diffusion exchanges of oxygen and carbonic acid. Dr. Dalton, better informed upon this subject, avoids this error.

In the next chapter, (thirteen,) on animal heat, Dr. Dalton concludes that this "phenomenon results from the simultaneous activity of many different processes, taking place in many different organs, and dependent, undoubtedly, on different chemical changes in each one. * * * The numerous combinations and decompositions which follow each other incessantly during the nutritive process, result in the production of an internal or vital heat, which is present in both animals and vegetables, and which varies in amount in different species, in the same individual at different times, and even in different parts and organs of the same body."

The next three chapters treat of the circulation, secretion, and excretion, and terminate the section on nutrition. The chapter on the circulation is graphically written, and handsomely and judiciously illustrated. Dr. Dalton adopts Harvey's theory of the movements of the heart, and follows very closely the admirable description of these movements long ago announced to the world by that eminent and truthful observer, who, after "having frequent recourse to vivisections, employing a variety of animals for the purpose, and collating numerous observations, thought that he had attained to the truth, and discovered both the motion and the use of the heart," though at first he "found the task so truly arduous and so full of difficulties, that he was almost tempted to think with Fracastorius," as he informs us, "that the motion of the heart was only to be comprehended by God." Our author having satisfied himself, by positive experiment, of the truth of the systolic theory, takes no notice of that other theory—the diastolic—which attributes the impulse of the heart to dilatation of the ventricles, and which, though generally regarded as a modern view, is in reality the older theory, as may be seen from the following paragraph from Harvey's writings:—

"It is generally believed that when the heart strikes the breast, and the pulse is felt without, the heart is dilated in its ventricles and is filled with blood; but the contrary of this is the fact, and the heart, when it contracts, (and the shock is given,) is emptied. Whence the motion, which is generally regarded as the diastole of the heart, is in truth its systole."

From this hasty survey of the first section of his work, it will be seen that Dr. Dalton very philosophically treats of nutrition not as one of several functions, but as a highly complex process, made up of many and diverse acts, whose great object is not only to provide for the reception of aliment, and its assimilation or conversion into the various tissues and organs, but also to preserve these parts from disease by the constant removal of effete particles by exuration and excretion. Dr. Bennett also regards and treats the function of nutrition as a compound one. He divides it into five stages, viz. :—1st. The introduction into the stomach and intestinal canal of appropriate alimentary matters. 2d. The formation from these of a nutritive fluid, the blood, and the changes it undergoes in the lungs. 3d. Passage of fluid from the blood to be transformed into the tissues. 4th. The disappearance of the transformed tissues, and their reabsorption into the blood. 5th. The excretion of these effete matters from the body in various forms and by different channels. "We believe," he very correctly observes, "that it is only by understanding nutrition in this enlarged sense, that we can obtain a correct explanation of the dependence of one process upon another, as well as of those important affections which may appropriately be called diseases of nutrition."

The second section of Dr. Dalton's work is devoted to the consideration of the nervous system. Dr. Bennett also treats of the "function of innervation," directly after that of nutrition. Dr. Dalton divides his account of the nervous system into six chapters, which severally treat of the general character and functions of the nervous system; nervous irritability, and its mode of action; the spinal cord, brain, cranial nerves, and great sympathetic. As far as it goes, this section is admirable; but as it does not go as far as it should, we are compelled to say that on this account, and on this account only, it is also objectionable. For the omissions are sufficiently grave and numerous to impair seriously the value of this work as a physiological *text-book*. All that is said about the physiology of the senses, is contained in about thirty lines of the chapter on the cranial nerves. The mechanism of the eye and ear, the physiology of vision, audition, taste, and touch, are altogether omitted. The brief description of the minute structure of the nervous centres contains no allusion to the recent laborious researches of Jacubowitsch and Lenhossek. The labors of the former, particularly, are well calculated to throw light upon the pathology of some of the most obscure neuroses, as well as to clear up many of the dark points in the physiology of the nervous system. These investigations were noticed by Flourens, before the *Académie des Sciences*, in the most eulogistic manner. In a physiological treatise designed for students and physicians, such omissions are altogether inexcusable. Even in the physiologico-pathological primer of Dr. Bennett, (we say primer, for it claims to be, and really is nothing more,) the special senses receive as much space as their importance and the size of the volume require. It is true, that in his preface, Dr. Dalton says that "physiological questions, which are in an altogether unsettled state, as well as purely hypothetical topics, have been purposely avoided, as not coming within the plan of this work, nor as calculated to increase its usefulness." But this apologetic paragraph does not apply to the omissions in question, for many points connected with the physiology of the senses, so far from being "unsettled and hypothetical," are as well established as any in the science, better in fact, as we have already seen, than the glycogenic function of the liver, to which a separate chapter has been devoted by our author. The structure and functions of the pneumogastric nerve and great sympathetic system, are discussed in a manner and to an extent much more in accordance with their importance. In the pages devoted to their consideration, the student will find all that is positively known concerning these portions of the nervous system.

In section third, reproduction is considered in an elaborate, thoughtful, and eminently philosophical manner. Though the last function treated of in the book, it is by far the most prominent. The author appears to have thrown all his energies into the execution of this part of his work. Nowhere does he appear to greater advantage as a physiologist. This, indeed, was to be anticipated, when we call to mind his former labors in this field, "on the corpus luteum of menstruation and pregnancy." We have already specified in the earlier part of this article the various details which constitute the subject-matter of this section. Our exhausted space will not permit us to enter upon the consideration of these details, although all of them are valuable, and some highly interesting, in consequence of involving views at variance with certain generally received doctrines.

Thus far we have considered Dr. Dalton's work as a *text-book* of physiology, "*designed,*" as the title-page has it, "*for the use of Students and Practitioners of Medicine.*" Regarding it in this light, regarding it as a book offered to the medical public for consultation upon the numerous facts and details of physiology, we have been compelled, in behalf of both student and practitioner, to point out its deficiencies, which, as already shown, are those of omission chiefly, rather than commission. Were we, however, to pause in our criticism here, we should be guilty of injustice to the author and his work, and should convict ourselves of a glaring want of discrimination. Let us, then, view the treatise before us as coming from the hands not of a mere compiler, but of an original observer, and let us look upon that treatise not as a repertory or magazine of physiological details, which it is far from being, but as a general survey or exposition of the science as a whole, by one of its laborious votaries. Then we discover that the deficiencies which we have noted become consistencies, and that many of the omissions about which we, in common with others, have been caviling per necessity, become evidences of the skill with which the author has selected and grouped his facts into a complete and harmonious whole. Then we discover that the facts and theories necessary to be presented in his exposition of the course of life in man, were laboriously subjected in the crucible of experiment to a rigid analysis, and all that was in any way calculated to strengthen this exposition was retained, while all that was likely to obscure or clog it was cast aside. Herein lies the secret of the remarkable consistency of this book. Dr. Dalton never loses sight of the fact that he is uttering to the world his opinion or theory of physiology as a unit. Hence the facts of one chapter in his work never, as far as we can see, conflict with those of another, as would almost certainly have been the case had the work been a mere compilation instead of a well-digested treatise. Convinced, for example, of the correctness of the systolic theory of the cardiac movements, he teaches it positively, and never alludes to the diastolic theory. Believing that oxygen exists in the blood in a state of solution, and not in a state of intimate chemical combination, he takes good care not to rank this substance among the proximate principles. Satisfied, from his own observations and experiments, that the red globules of the blood are as permanent anatomical forms as muscular fibres and nervous filaments, and that they do not perish bodily in any part of the circulation, he ignores the idea of the transformation of globulin into hæmatoidin or hæmato-crystallin, appearing to believe with Robin and Verdeil, that the blood contains no crystallizable proximate principle of organic origin.

From different parts of his work, we might readily adduce other examples of a similar scope, all going to show that Dr. Dalton has treated his subject in a highly original manner, and that, with much independence of thought, he has rejected some theories ordinarily received, and accepted others less well known, and that he has assigned to many facts new values, elevating some and depressing others in the scale of physiological importance. He makes no copious references to authorities, and indulges in no vain parade of the facts discovered and theories advanced by different physiologists. On the contrary, in every page of his book we find unmistakable evidence that the author is giving us not the views of others, but his own opinions, the value and reliability of which he has conscientiously tested in the most careful and pains-taking manner. His work comes to us, therefore, with an authoritative value, which no compilation, however encyclopedic, or however well arranged, can possess. In conclusion, then, if we were asked our opinion concerning this volume, we should say without hesitation that, as a comprehensive book of reference for the facts of physiology, it is faulty, and in some respects is inferior to the well-known works of Carpenter, Valentin, Kirkes and others; but as a compendious and graphic treatise, as a lucid and concise exposition or summary of the leading or fundamental principles of the science of life in health, it is not only superior to the works just mentioned, but in many particulars is perhaps unequaled. The "getting up" of the work is in every respect admirable. Paper of a superior quality, excellent typography, and numerous wood-cuts, as novel in design as they are handsome in execution, give to it an attractiveness which speaks loudly in praise of the liberality and enterprise of the publishers, who have evidently been determined to clothe the valuable and instructive teachings of the author in a most beautiful and appropriate dress.

The elementary volume of Dr. Bennett is also deserving of commendation—not so much for the number and variety of its facts, as for the clinical spirit with which the truths of physiology are inculcated. Dr. Bennett, as we have already intimated, is not so much a physiologist as a physiological physician—a practitioner who finds in physiology the scientific basis of his art. The character of his mind, in this respect, is reflected from every page of his *Outlines*. He neglects no opportunity to remind the student of the exact medical bearings of the more important and useful facts of physiology. This is well shown in the remarks with which the section on nutrition terminates, and especially in the third part of the work which is devoted to "pathological physiology," and which contains, in a condensed form, many of those peculiar views of the author concerning inflammation, blood-letting, etc., which have recently involved him in such an animated controversy with Alison and others, of Edinburgh.

